# One-Year Outcomes After ICU Discharge in Patients Aged 80 Years and Older: Functional and Clinical Factors Associated with Mortality in a Retrospective Observational Cohort

**DOI:** 10.3390/jcm15145324

**Published:** 2026-07-08

**Authors:** Derful Gülen, Serpil Ekin, Buket Özyaprak, Ilkay Ceylan

**Affiliations:** Department of Anesthesiology and Reanimation, Bursa Yüksek Ihtisas Training and Research Hospital, University of Health Sciences, Bursa 16310, Türkiye

**Keywords:** intensive care unit (ICU), post-discharge mortality, comorbidity, post-ICU care, very elderly adults

## Abstract

**Objective:** This study aimed to evaluate clinical and functional factors associated with one-year mortality after intensive care unit (ICU) discharge among ICU survivors aged 80 years and older, with particular emphasis on comorbidity burden and post-discharge dependency. **Methods:** This retrospective, single-center observational study included 131 ICU survivors aged ≥ 80 years who were discharged alive from the ICU between January 2023 and April 2025. Patients were categorized according to one-year survival status after ICU discharge as survivors (n = 63) and non-survivors (n = 68). Demographic characteristics, comorbidities, acute illness severity scores, ICU- and hospital-related variables, discharge characteristics, post-discharge care requirements, respiratory support status, and nutritional routes were analyzed. Multivariable logistic regression analysis was performed to identify factors independently associated with one-year mortality. **Results:** The one-year mortality rate after ICU discharge was 51.9%. Non-survivors were significantly older than survivors (85.41 ± 3.85 vs. 84.30 ± 4.26 years, *p* = 0.038). The Charlson Comorbidity Index (CCI) was significantly higher in non-survivors (5.69 ± 2.27 vs. 4.08 ± 2.07, *p* < 0.001) and remained independently associated with one-year mortality (OR = 1.432, 95% CI: 1.107–1.851; *p* = 0.006). Additionally, post-discharge care requirement was independently associated with increased mortality (OR = 6.35, 95% CI: 2.83–14.21; *p* < 0.001), while the presence of spontaneous breathing was associated with lower odds of mortality (OR = 0.32, 95% CI: 0.15–0.66; *p* = 0.002). ICU and hospital lengths of stay were longer in non-survivors, and vasopressor use was more frequent in this group. Survivors were more likely to be discharged home, were less likely to require post-discharge care, and had higher rates of spontaneous breathing. In contrast, non-survivors more frequently required post-discharge care and oxygen concentrator support. **Conclusions:** Among ICU survivors aged 80 years and older who were discharged alive from the ICU, one-year mortality after discharge was strongly associated with comorbidity burden and post-discharge dependency indicators. These findings highlight the importance of structured post-ICU follow-up, functional assessment, and individualized care planning for very elderly ICU survivors.

## 1. Introduction—Objective

The global elderly population is growing rapidly, placing an increasing burden on healthcare systems. According to the United Nations’ *World Population Prospects 2024* report, the proportion of elderly individuals in the total population is expected to rise steadily in the coming decades, further highlighting the trend of global aging [1]. This demographic transition significantly impacts healthcare planning and resource allocation. As the elderly population increases, clinical research and health policy efforts targeting this age group have also accelerated. With advancing age, the burden of comorbidities rises, physiological reserves diminish, and functional capacity tends to decline. As a result, older adults exhibit an increased need for healthcare services, including the use of intensive care units (ICUs) [2,3].

Recent geriatric-focused studies on ICU patients have begun to treat individuals aged 80 years and older as a distinct subgroup. In the present study, patients aged ≥ 80 years who survived ICU discharge are referred to as very elderly ICU survivors. This age group has gained attention due to its unique clinical characteristics and prognosis, making it a focal point for research. As a result, studies focusing on this population are increasingly represented in the literature [4,5,6].

However, data regarding long-term clinical outcomes for elderly patients discharged from the ICU remain limited. This highlights the necessity for post-discharge follow-up and the development of care strategies that extend beyond the acute treatment phase. Tracking clinical progression, survival rates, and quality of life after ICU discharge in this population can inform efficient resource allocation, enhance the organization of long-term care services, and support ethically sound end-of-life decisions.

In this context, variables reflecting comorbidity burden, acute illness severity, and post-discharge clinical dependency have been consistently identified as key factors associated with long-term outcomes in very elderly ICU survivors and therefore constitute the conceptual framework for the variable selection in the present study.

We hypothesized that higher comorbidity burden, markers of clinical severity, and post-discharge functional dependency would be associated with increased one-year mortality in patients aged ≥ 80 years after ICU discharge. The results are expected to support the development of elderly-specific healthcare policies, reinforce multidisciplinary care strategies, and enhance clinical decision-making processes.

Although the association between comorbidity burden and poor outcomes in very elderly ICU survivors may appear clinically intuitive, the prognostic role of post-discharge dependency indicators remains less clearly defined in very elderly ICU survivors. In particular, the combined evaluation of comorbidity burden, discharge destination, post-discharge care requirement, respiratory independence, and nutritional route may provide additional clinical insight beyond traditional acute illness severity scores. Therefore, the present study aimed not only to confirm known mortality-associated factors, but also to highlight the importance of post-ICU transitional care in risk stratification for one-year outcomes among patients aged 80 years and older.

## 2. Materials and Methods

### 2.1. Study Design and Ethical Approval

This retrospective, single-center observational study was conducted in the intensive care unit of the Department of Anesthesiology and Reanimation at University of Health Sciences Bursa Yüksek Ihtisas Training and Research Hospital.

Ethics approval was obtained from the Clinical Research Ethics Committee of University of Health Sciences Bursa Yüksek Ihtisas Training and Research Hospital (Approval No: 2024-TBEK 2025/05-16).

As the study involved only retrospective analysis of anonymized routine medical records, informed consent from participants was not required, and the need for individual consent was waived by the ethics committee.

**Human Ethics and Consent to Participate declarations:** Ethics approval was obtained; informed consent was waived.

**A Clinical Trial Number was not applicable**, as this was a retrospective and non-interventional study.

All procedures were conducted in accordance with institutional guidelines and the ethical principles of the Declaration of Helsinki, with full respect for patient confidentiality.

### 2.2. Study Population

No formal sample size calculation was performed, as this was a retrospective observational study; instead, all consecutive patients aged 80 years and older who were admitted to the ICU during the study period were screened for eligibility.

The study included patients aged 80 years and older who were admitted to the ICU between 1 January 2023 and 30 April 2025. During this period, a total of 394 patients aged ≥ 80 years were identified. Patients who died during their ICU stay were excluded, as the primary aim of the study was to evaluate one-year outcomes after ICU discharge. Therefore, the study cohort represents very elderly ICU survivors who were discharged alive from the ICU, rather than all patients aged 80 years and older admitted to the ICU during the study period.

The inclusion criteria were complete documentation of key clinical data during the ICU stay and clearly ascertainable survival status one year after discharge. Only the first ICU admission per patient was considered. Patients with incomplete records or repeated ICU admissions were excluded. This ensured that each patient contributed a single, independent observation and avoided within-patient clustering. After applying these exclusion criteria, 131 patients who were discharged alive from the ICU were eligible for inclusion and were analyzed. According to their survival status one year after discharge, patients were categorized into two groups: survivors (Group 1) and non-survivors (Group 2).

### 2.3. Study Outcomes

The primary endpoint was defined as all-cause one-year survival status after ICU discharge. Other clinical parameters, including ICU and hospital length of stay, need for mechanical ventilation, vasopressor and renal replacement therapy, ICU readmission after discharge from the first ICU admission, discharge destination, post-discharge care requirements, level of respiratory support, and route of nutrition, were collected and analyzed as descriptive and comparative variables in relation to the primary outcome.

### 2.4. Data Collection

Data were retrospectively extracted from the hospital information management system and individual patient records. Demographic variables included age and sex. Clinical data encompassed the presence of multiple trauma, cerebrovascular pathology, malignancy, pulmonary and cardiac conditions, chronic organ failure, and neuromuscular diseases.

One-year survival status after ICU discharge was determined by reviewing hospital electronic medical records and cross-referencing with the national death registry (e-Nabız system), which provides updated mortality information based on official government data. Follow-up completeness was assessed by verifying one-year survival status for all patients included in the final cohort. Patients whose one-year survival status could not be reliably ascertained were excluded during cohort selection. Therefore, mortality ascertainment was complete for all 131 patients included in the final analysis. However, detailed information regarding the specific causes of death during follow-up was not consistently available in the medical records or registry data. Therefore, the present study evaluated all-cause one-year mortality rather than cause-specific mortality.

The comorbidity burden was evaluated using the Charlson Comorbidity Index (CCI), and nutritional status was assessed with the Nutritional Risk Screening 2002 (NRS-2002) score. Additionally, body mass index (BMI) was calculated using patients’ weight and height at the time of discharge.

In the Turkish healthcare system, Do Not Attempt Cardiopulmonary Resuscitation (DNACPR) orders are not legally recognized as part of routine clinical documentation. Therefore, data on treatment escalation planning, therapeutic limitations, and goals-of-care decisions could not be systematically assessed in this study.

### 2.5. Assessment of Clinical Severity and ICU Variables

To determine the severity of critical illness, both the APACHE II (Acute Physiology and Chronic Health Evaluation II) and SOFA (Sequential Organ Failure Assessment) scores were calculated within the first 24 h of ICU admission, allowing for a detailed evaluation of organ dysfunction and overall clinical severity.

ICU-related variables collected for analysis included the length of ICU stay and total hospital stay, the use of mechanical ventilation and vasopressors, tracheostomy during the ICU stay, the need for renal replacement therapy, ICU readmission after discharge from the first ICU admission, ICU-acquired infections, and the occurrence of septic shock.

ICU-acquired infections were defined as clinically and/or microbiologically documented infections first recognized ≥48 h after ICU admission, consistent with standard healthcare-associated infection surveillance definitions [7].

Septic shock was defined according to the Sepsis-3 criteria as persistent hypotension requiring vasopressors to maintain mean arterial pressure ≥ 65 mmHg and a serum lactate level > 2 mmol/L despite adequate fluid resuscitation [8].

In the post-discharge period, the following parameters were recorded: discharge destination, post-discharge care requirement, respiratory support status, and nutritional route. All data were retrospectively analyzed using standardized data collection forms. Baseline functional independence, pre-ICU place of residence, pre-existing care needs, formal cognitive status, and validated frailty scores were not routinely documented in the retrospective medical records and therefore could not be included in the analysis.

Post-discharge care requirement was defined as the need for ongoing professional or institutional care after ICU discharge, including transfer to a palliative care unit, nursing home, or the need for formal caregiver support documented in the discharge records. Patients discharged home without documented post-discharge care requirement were classified as not requiring post-discharge care.

Spontaneous breathing was defined as breathing without invasive mechanical ventilation, tracheostomy-dependent ventilation, or home mechanical ventilation support at ICU discharge. Patients who required only supplemental oxygen or oxygen concentrator support without mechanical ventilatory assistance were classified as having spontaneous breathing.

### 2.6. Statistical Analysis

All data were analyzed using IBM SPSS Statistics for Windows, Version 26.0 (IBM Corp., Armonk, NY, USA). A 95% confidence interval was applied to all statistical tests. All statistical tests were two-sided, and a uniform significance threshold of *p* < 0.05 was applied to all comparisons and regression analyses. Exact *p*-values were reported where possible, with values below 0.001 reported as *p* < 0.001. Continuous variables were expressed as mean ± standard deviation (mean ± SD), while categorical variables were presented as counts (n) and percentages (%).

The normality of continuous variables was assessed using the Shapiro–Wilk test and visual inspection of histograms and Q–Q plots. Group comparisons were performed using Student’s *t*-test for normally distributed variables or the Mann–Whitney U test for non-normally distributed variables, as appropriate. All categorical variables were compared using Fisher’s exact test.

Multivariable logistic regression analysis was performed to identify factors independently associated with one-year mortality among elderly ICU survivors. Variables included in the multivariable logistic regression model were selected based on clinical relevance and univariate analysis results and included age, APACHE II score, Charlson Comorbidity Index, mechanical ventilation, vasopressor support, renal replacement therapy, post-discharge care requirement, and spontaneous breathing status. The Hosmer–Lemeshow goodness-of-fit test and area under the ROC curve (AUC) were used to evaluate model performance. Multicollinearity was assessed using the variance inflation factor (VIF), and values < 5 were considered acceptable.

Given the relatively limited sample size and number of outcome events, the multivariable models were constructed using a restricted number of clinically relevant variables to reduce the risk of overfitting. Model performance was assessed using the Hosmer–Lemeshow goodness-of-fit test, the area under the receiver operating characteristic curve with 95% confidence intervals, and multicollinearity diagnostics using variance inflation factors. Internal validation was additionally performed using bootstrap resampling to estimate optimism-corrected discrimination. External validation was not possible because of the single-center retrospective design.

In addition to the primary multivariable logistic regression model, selected post-discharge variables were included in extended analyses to explore their association with long-term mortality. Post-discharge variables, including post-discharge care requirement and spontaneous breathing status, were included in the multivariable model for prognostic risk stratification rather than causal inference. These variables were available at or immediately after ICU discharge and were considered clinically relevant discharge-phase indicators of patient vulnerability. However, because such variables may partly reflect consequences of the acute ICU course and may lie on the pathway between baseline illness severity and long-term mortality, their inclusion may introduce overadjustment. Therefore, the associations involving post-discharge variables were interpreted as prognostic and hypothesis-generating rather than causal.

To increase the clinical interpretability of post-discharge dependency and to address the need for additional clinically relevant indicators, an exploratory post-discharge vulnerability analysis was performed using available discharge-phase variables. These variables included post-discharge care requirement and non-home discharge destination, defined as discharge to palliative care or nursing home. One-year mortality was defined as death occurring within 365 days after ICU discharge. The associations of these discharge-phase indicators with one-year mortality were evaluated using Fisher’s exact test, and unadjusted odds ratios with 95% confidence intervals were calculated. This analysis was considered exploratory and hypothesis-generating.

Survival probabilities over the one-year follow-up period were estimated using the Kaplan–Meier method, and differences between survival curves were assessed using the Log-rank (Mantel–Cox) test. Time-to-event was calculated from the date of ICU discharge to the date of death or censoring at 365 days. Patients who were alive for one year were censored at 365 days. Because one-year survival status was ascertainable for all patients in the final cohort, no patients were censored before 365 days because of loss to follow-up. Patients were stratified into subgroups based on the Charlson Comorbidity Index (CCI) score (<5 vs. ≥5) and post-discharge care requirement.

Because the outcome also involved time-to-death within one year after ICU discharge, multivariable Cox proportional hazards regression analysis was additionally performed. Follow-up time was calculated from ICU discharge to death or censoring at 365 days. Patients alive at one year were censored at 365 days. The Cox model included age, Charlson Comorbidity Index, ICU length of stay, mechanical ventilation duration, vasopressor use, and post-discharge care requirement. Results were reported as adjusted hazard ratios with 95% confidence intervals. The Cox regression results were visualized using a forest plot.

Missing data were assessed before analysis. Patients with missing or incomplete key clinical data were excluded during cohort selection. Therefore, the final analytic cohort consisted of 131 patients with complete data for the variables included in the descriptive, comparative, multivariable logistic regression, and Cox proportional hazards regression analyses. The proportion of missing data for analyzed variables in the final cohort was 0%; therefore, multiple imputation was not performed.

## 3. Results

A total of 394 patients aged 80 years and older were admitted to the intensive care unit (ICU) during the study period. Of these, 243 patients died during the ICU stay, corresponding to an ICU mortality rate of 61.7%. Because the primary objective of the study was to evaluate outcomes after ICU discharge, patients who died during their ICU stay were excluded from the post-discharge survival analysis. Additional exclusions included patients with missing or incomplete clinical data (n = 9) and those with repeated ICU admissions during the study period (n = 11).

**Figure 1:**
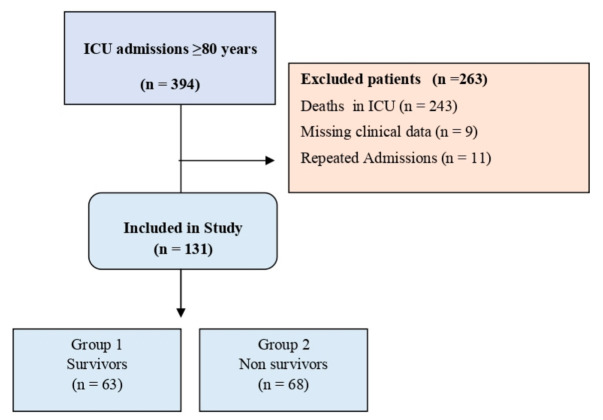
Flow diagram of patient selection and study cohort formation.

After application of these exclusion criteria, 131 patients who were discharged alive from the ICU with complete clinical data and a clearly ascertainable one-year survival status were included in the final analysis. No missing data were present for the variables analyzed in the final cohort; therefore, all 131 patients were included in the descriptive, comparative, multivariable logistic regression, and Cox proportional hazards regression analyses. One-year survival status was successfully ascertained for all patients in the final cohort, corresponding to 100% follow-up completeness for the primary outcome. At the end of the one-year follow-up period after ICU discharge, 63 patients (48.1%) were alive, whereas 68 patients (51.9%) had died. Based on survival status over one year, patients were categorized into survivors (Group 1) and non-survivors (Group 2). The patient selection process is summarized in Figure 1.

The demographic and clinical characteristics of the patients are summarized in Table 1. A statistically significant difference in age was observed between the groups, with non-survivors being older than survivors (*p* = 0.038). In addition, the CCI was significantly higher in non-survivors compared with survivors (*p* < 0.001). Body mass index (BMI) was significantly higher in the survivor group (27.86 ± 3.80 kg/m^2^) than in the non-survivor group (26.41 ± 4.05 kg/m^2^; *p* = 0.044). Other baseline demographic and clinical variables were comparable between the groups.

In the multivariable logistic regression model, the Charlson Comorbidity Index, post-discharge care requirement, and spontaneous breathing were found to be associated with one-year mortality (Table 2). Higher CCI and the need for post-discharge care were associated with increased mortality risk, whereas the presence of spontaneous breathing was associated with reduced mortality. Other variables, including age, APACHE II score, mechanical ventilation, vasopressor use, and renal replacement therapy, were not significantly associated with one-year mortality in this model. The logistic regression model demonstrated acceptable discrimination, with an area under the ROC curve of 0.75 (95% CI: 0.66–0.83). Calibration was acceptable according to the Hosmer–Lemeshow goodness-of-fit test (χ^2^ = 9.25, df = 8, *p* = 0.322). No concerning multicollinearity was observed among the included variables, with variance inflation factor values ranging from 1.02 to 1.59. Bootstrap internal validation showed an optimism-corrected AUC of 0.70, suggesting that model performance should be interpreted cautiously in the context of the relatively small sample size.

**Figure 2:**
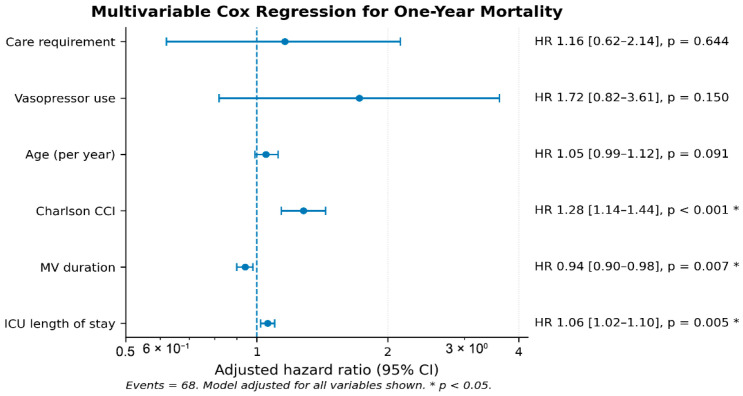
Forest plot of adjusted hazard ratios and 95% confidence intervals for predictors of one-year mortality after ICU discharge based on the multivariable Cox proportional hazards regression model. Follow-up was defined from ICU discharge to death or censoring at 365 days. The vertical dashed line indicates HR = 1. HR: hazard ratio; CI: confidence interval; ICU: intensive care unit.

An additional multivariable Cox proportional hazards regression analysis was performed to account for time-to-death within one year after ICU discharge (Figure 2). In this model, higher Charlson Comorbidity Index was significantly associated with increased one-year mortality hazard after ICU discharge (HR = 1.28, 95% CI: 1.14–1.44; *p* < 0.001). Longer ICU length of stay was also associated with increased mortality hazard (HR = 1.06, 95% CI: 1.02–1.10; *p* = 0.005). Mechanical ventilation duration showed an inverse association with mortality hazard (HR = 0.94, 95% CI: 0.90–0.98; *p* = 0.007), which should be interpreted cautiously in the context of the retrospective design, survivor selection, and potential residual confounding. Age, vasopressor use, and post-discharge care requirement were not significantly associated with mortality hazard in this Cox model. The adjusted hazard ratios and 95% confidence intervals from the Cox model are presented in Figure 2 as a forest plot.

**Figure 3:**
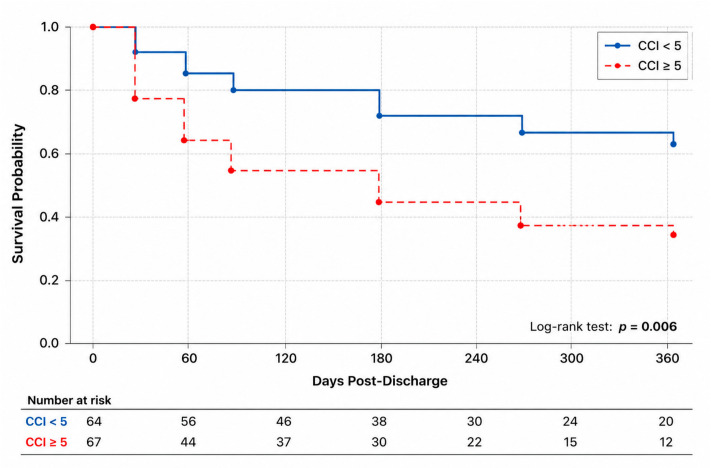
Kaplan–Meier survival curves for one-year mortality following ICU discharge, stratified by the Charlson Comorbidity Index (CCI). The red dashed line represents patients with a high comorbidity burden (CCI ≥ 5), demonstrating significantly higher mortality compared to the low-burden group (CCI < 5, blue line).

Kaplan–Meier survival analysis was performed according to comorbidity burden. In the Kaplan–Meier analyses, death within 365 days after ICU discharge was considered the event, and patients alive at one year were censored at 365 days. A significant difference in one-year survival was observed between patients with CCI ≥ 5 and those with CCI < 5, with lower survival probability in the higher CCI group (Log-rank *p* = 0.006; Figure 3).

The ICU and hospital length of stay, along with treatment-related clinical characteristics of the groups, are presented in Table 3. The difference in ICU length of stay between the groups was borderline statistically significant. The mean ICU length of stay was 11.22 ± 12.47 days in the survivor group and 17.59 ± 19.85 days in the non-survivor group (*p* = 0.059). Hospital length of stay was significantly longer in the non-survivor group, with a mean duration of 26.00 ± 25.17 days compared with 17.95 ± 15.05 days in the survivor group (***p* = 0.043**). Use of vasopressors was significantly higher in the non-survivor group. Vasopressor therapy was administered to 7 patients (11.1%) in Group 1 and 20 patients (29.4%) in Group 2 (***p* = 0.016**). Tracheostomy was performed in 24 of 131 ICU survivors (18.3%). The frequency of tracheostomy was numerically higher in non-survivors than in survivors, although this difference did not reach statistical significance (23.5% vs. 12.7%; *p* = 0.120).

Significant differences were observed between the groups in terms of discharge destination and post-discharge care requirements (Table 4). Discharge to home was more frequent in the survivor group compared with the non-survivor group (88.9% vs. 69.1%; ***p* = 0.013**). Conversely, discharge to a palliative care unit was more common in the non-survivor group (26.5% vs. 11.1%; ***p* = 0.035**). Furthermore, the need for post-discharge care was significantly higher among non-survivors compared with survivors (57.4% vs. 17.5%; ***p* < 0.001**).

**Figure 4:**
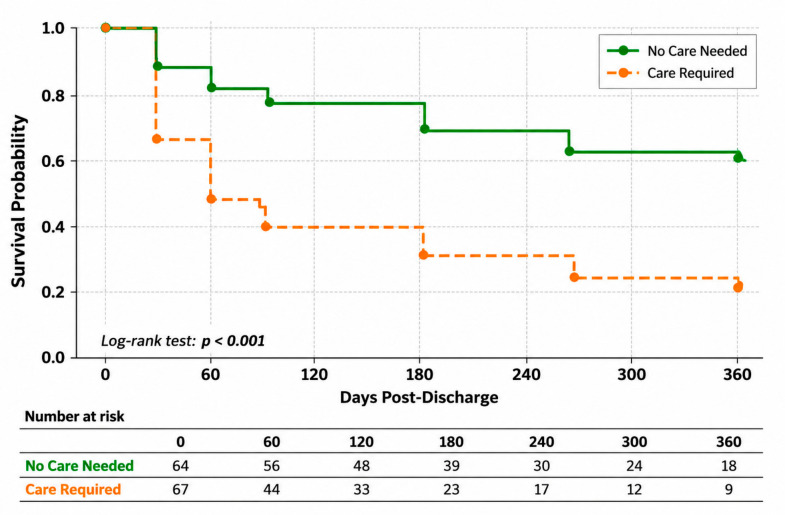
Kaplan–Meier survival estimates based on post-discharge care requirements.

Kaplan–Meier survival analysis was performed according to post-discharge care requirement. Patients were analyzed as two groups: those with and without post-discharge care requirement. A significant difference in one-year survival was observed between the groups, with lower survival probability in patients with post-discharge care requirement (Log-rank *p* < 0.001; Figure 4).

Post-discharge care requirement served as a pragmatic marker of post-discharge dependency, showing a significant divergence in survival probability compared with the independent group from the early post-discharge period.

Table 5 presents a comparison of post-discharge respiratory characteristics between the groups. The presence of spontaneous breathing was significantly higher in the survivor group compared with the non-survivor group (69.8% vs. 42.7%; ***p* = 0.003**). In contrast, the need for an oxygen concentrator was significantly more frequent in the non-survivor group (33.8% vs. 17.5%; ***p* = 0.046**).

To further evaluate post-discharge dependency, an exploratory post-discharge vulnerability analysis was performed using discharge-phase indicators, including post-discharge care requirement and non-home discharge destination. Overall, 50 patients required post-discharge care, and 28 patients were discharged to a non-home setting, including palliative care or nursing home. In this exploratory unadjusted analysis, post-discharge care requirement was significantly associated with one-year mortality (OR = 6.35, 95% CI: 2.83–14.21; *p* < 0.001). Similarly, non-home discharge destination was also associated with increased odds of one-year mortality (OR = 3.57, 95% CI: 1.40–9.14; *p* = 0.010) (Table 6).

## 4. Discussion

The post-discharge clinical course of elderly individuals following intensive care unit (ICU) treatment is gaining increasing importance in healthcare planning and in anticipating long-term care needs. For patients aged 80 and older, the burden of comorbidities, reduced functional capacity, and continued need for care or respiratory support after discharge are key factors associated with post-ICU prognosis [6,9,10].

Importantly, the present study focused specifically on patients who survived to ICU discharge. Therefore, the findings should be interpreted as reflecting post-discharge prognosis among very elderly ICU survivors rather than outcomes among all very elderly patients admitted to the ICU.

The main contribution of this study is the demonstration that one-year mortality in very elderly ICU survivors is associated not only with comorbidity burden but also with clinically observable post-discharge dependency indicators. Although higher CCI is an expected factor associated with poor outcome, the associations of post-discharge care requirement and spontaneous breathing status highlight the prognostic importance of the transition period after ICU discharge. This finding suggests that discharge-phase variables may help identify vulnerable patients who require closer follow-up, structured rehabilitation, and individualized care planning.

The ICU mortality rate among screened patients was high, with 243 of 394 patients dying before ICU discharge. This finding highlights the extreme vulnerability of very elderly patients requiring intensive care and underscores the importance of distinguishing ICU mortality from post-discharge mortality. Because the present analysis focused only on patients who survived ICU discharge, the reported one-year mortality rate should be interpreted as post-discharge mortality among ICU survivors rather than overall mortality among all very elderly ICU admissions.

Mortality in this age group is influenced by factors such as pre-ICU functional status, ICU length of stay, the nature of the discharge destination, respiratory support requirements, and nutritional methods [4,5,6]. However, studies evaluating both ICU-related and post-discharge variables simultaneously in patients aged 80 years and older remain limited [11].

In our study, the mean age was found to be significantly higher in the non-survivors group (Table 1). This observation is in line with previous studies of very elderly ICU populations. Advanced age is an important variable associated with poorer post-ICU survival due to reduced physiological reserve and increased comorbidity burden. Nevertheless, age alone is not a strong prognostic marker; instead, it interacts with factors such as frailty, functional status, and comorbidities to determine mortality risk. Andersen et al. reported that age was associated with mortality in patients aged 80 years and older, but its independent effect remained limited in multivariable analyses [5]. The VIP2 study by de Lange et al. reported that age may influence mortality when evaluated together with premorbid condition and functional capacity [6]. In our study, however, age did not retain statistical significance in the multivariable model (Table 2), suggesting that its effect on long-term mortality is mediated through comorbidity burden and functional status rather than acting independently.

Regarding BMI, values measured at ICU admission were significantly higher in the survivor group (Table 1). However, mean BMI values in both groups were within the overweight range. Therefore, this finding should be interpreted with caution. In very elderly patients, lower admission BMI may reflect reduced nutritional reserve or underlying frailty rather than a protective effect of higher body weight. Accordingly, this finding should be considered hypothesis-generating rather than indicative of a direct causal relationship, particularly in the absence of multivariable significance.

Although NRS 2002 scores did not significantly differ between survivors and non-survivors, this finding highlights the limitations of the tool in predicting long-term outcomes. NRS focuses primarily on short-term nutritional risk and does not account for chronic malnutrition, sarcopenia, or frailty, which are highly prevalent in elderly ICU populations. These factors, though unmeasured in our study, may contribute significantly to long-term mortality and may explain the observed difference despite comparable nutritional scores at discharge.

CCI emerged as one of the variables most strongly associated with mortality in our study. The mean CCI was significantly higher in the non-survivor group, indicating a greater overall comorbidity burden among patients who died within one year after ICU discharge (Table 1). Prior studies have similarly shown that higher CCI scores have been associated with reduced post-ICU survival and significantly increased mortality rates [4,5]. With increasing comorbidity burden, the rates of invasive interventions and treatment intensity tend to decrease, while organ dysfunction and hospital mortality increase, particularly in very elderly patients [4].

Kaplan–Meier survival analysis further supported these findings by demonstrating a clear separation in survival curves according to CCI levels (Figure 3). Patients were stratified using a clinically relevant threshold (CCI < 5 vs. ≥5), which has been previously utilized to distinguish low- and high-risk comorbidity burden groups in the literature [12]. Patients with higher comorbidity burden exhibited a more pronounced decline in survival probability, particularly within the early post-discharge period, indicating that CCI is not only associated with overall mortality but also influences the temporal pattern of survival.

The additional Cox proportional hazards analysis further supported the prognostic importance of comorbidity burden over time, as higher Charlson Comorbidity Index was significantly associated with increased one-year mortality hazard. ICU length of stay was also associated with higher mortality hazard, suggesting that prolonged critical illness may reflect greater clinical complexity and vulnerability after ICU discharge. However, the inverse association observed for mechanical ventilation duration should be interpreted cautiously, as this may reflect survivor selection, treatment heterogeneity, or residual confounding rather than a protective effect.

This association was further supported by the multivariable logistic regression analysis, in which CCI remained independently associated with one-year mortality after adjustment for age, acute disease severity, and major ICU-related interventions (Table 2).

In our study, no significant difference was observed between survivors and non-survivors in terms of SOFA scores (Table 1). This finding suggests that acute physiological severity alone may not adequately reflect the risk of long-term mortality in very elderly ICU survivors. Similarly, APACHE II did not retain statistical significance in the multivariable model (Table 2), indicating that acute illness severity alone may be insufficient to explain long-term outcomes in this population.

These findings are also consistent with the results of the VIP study, a multicenter investigation, which reported that the prognostic value of acute illness severity scores may be limited in patients aged 80 years and older [13]. In this context, long-term prognosis in very elderly ICU patients appears to be more strongly influenced by factors such as premorbid functional status, frailty, and comorbidity burden.

The findings of the VIP study have emphasized that frailty, premorbid functional status, and geriatric vulnerability may provide prognostic information beyond chronological age and conventional acute illness severity scores in very elderly ICU patients. Frailty has been shown to be closely associated with short- and intermediate-term mortality, treatment intensity, and functional outcomes after critical illness. These observations are consistent with our findings, in which APACHE II and SOFA scores were not independently associated with one-year mortality, whereas comorbidity burden and post-discharge dependency indicators showed stronger prognostic relevance. Although a standardized frailty score was not available in our retrospective dataset, post-discharge care requirement, discharge destination, respiratory independence, and nutritional route may reflect aspects of vulnerability and dependency. However, these variables should not be considered substitutes for validated frailty instruments such as the Clinical Frailty Scale. The absence of direct frailty measurement may have resulted in residual confounding and should be considered when interpreting the associations observed in this study.

Recent literature published between 2023 and 2025 further supports the importance of frailty, functional trajectory, and post-ICU care needs in older ICU survivors. Jung et al. reported that older ICU survivors may experience persistently increased long-term mortality and altered functional trajectories compared with the general population, emphasizing that survival alone may not fully capture recovery after intensive care [14]. Similarly, Ueno et al. demonstrated that frailty was associated with long-term survival among ICU patients, supporting the need to consider frailty-related vulnerability when interpreting outcomes beyond the acute ICU period [15]. In addition, Saxena et al. highlighted the prognostic relevance of the Clinical Frailty Scale in critically ill patients, reinforcing the importance of validated frailty assessment tools in ICU populations [16]. Recent expert discussion has also emphasized the need to bridge the gap between ICU survival and long-term management of very old patients, including structured follow-up, functional assessment, and individualized post-ICU care planning [17]. These recent findings are consistent with our results and support the interpretation of post-discharge care requirement, discharge destination, respiratory independence, and nutritional route as pragmatic clinical indicators within a broader geriatric vulnerability framework.

Length of stay in the ICU is an important clinical indicator in elderly patients and may reflect complications, clinical complexity, and poorer prognosis. In our study, ICU length of stay was longer in the non-survivor group, and this difference was very close to reaching statistical significance (Table 3). In addition, hospital length of stay was significantly longer in the non-survivor group (Table 3). Previous studies have also reported an association between prolonged ICU or hospital stay and increased mortality in older populations [18,19]. This association, however, may be influenced by greater disease severity and clinical complexity rather than representing a direct causal effect.

The relatively high tracheostomy prevalence in this cohort may reflect the prolonged ventilatory needs and clinical complexity of very elderly ICU survivors; however, the lack of data on tracheostomy indication, timing, decannulation, and long-term dependence limits further interpretation.

Vasopressor use was associated with mortality in univariate analysis; however, this association did not remain significant after adjustment in the multivariable model (Table 2). Therefore, this finding should be interpreted with caution. Vasopressor requirement may reflect underlying illness severity and hemodynamic instability. Similarly, Sodhi et al. reported that vasopressor use was associated with increased mortality in very elderly ICU patients [20]. This finding suggests that vasopressor requirement may represent a marker of more severe and clinically unstable patients rather than serving as an independent prognostic factor.

The post-discharge care setting is an important clinical indicator that may reflect the level of clinical recovery and post-discharge dependency in very elderly ICU survivors. In our study, a significantly higher proportion of survivors were discharged to their homes, whereas this rate was notably lower in the non-survivor group (Table 4). Conversely, discharge to a palliative care unit was significantly more frequent among non-survivors. These findings suggest that discharge destination is closely related to patients’ clinical stability and overall well-being.

Patients referred to professionally supported environments, such as palliative care units or other institutional care settings, may represent those with greater post-discharge care dependency and clinical vulnerability. However, because functional status was not directly measured using validated instruments, discharge destination should be interpreted as an indirect clinical marker rather than a validated measure of functional dependency.

Similarly, a study by Ouchi et al. reported that discharge destination is closely associated with long-term survival and quality of life in older adults, noting that those discharged home had significantly longer survival than those referred to other care settings [21].

In line with the findings on discharge setting, post-discharge care requirement was also closely associated with mortality. In our study, post-discharge care requirement was significantly higher in the non-survivor group (Table 4). Moreover, post-discharge care requirement was independently associated with one-year mortality in the multivariable model (Table 2).

Kaplan–Meier survival analysis further demonstrated a significant divergence in survival curves according to post-discharge care requirement (Figure 4), with patients with post-discharge care requirement showing a markedly lower survival probability over time. These findings indicate that elderly ICU survivors are at high risk not only in terms of short-term mortality but also regarding post-discharge quality of life and dependency.

These findings provide time-dependent confirmation of the association between post-discharge dependency and mortality. Nevertheless, because functional status was not directly assessed using validated instruments such as the Barthel Index, Katz Activities of Daily Living scale, or Clinical Frailty Scale, post-discharge care requirement should be interpreted as a pragmatic marker of dependency rather than a direct measure of functional status. In addition, because baseline independence, pre-ICU residence, pre-existing care needs, and formal cognitive status were not available, post-discharge care requirement may partly reflect unmeasured pre-existing frailty or dependency rather than only new disability acquired during or after the ICU stay.

In response to the need for more clinically applicable post-ICU indicators, we also performed an exploratory post-discharge vulnerability analysis using post-discharge care requirement and non-home discharge destination as discharge-phase dependency markers. Both indicators were associated with one-year mortality in unadjusted analyses. These findings suggest that simple discharge-phase variables may help capture post-ICU vulnerability and identify very elderly ICU survivors at increased long-term risk. The strong association between care requirement and mortality further supports the concept that post-discharge dependency reflects the cumulative burden of chronic illness, respiratory impairment, and reduced physiological reserve. Although these indicators are not validated frailty or functional assessment tools, they may provide a pragmatic framework for identifying patients who require closer follow-up, rehabilitation planning, and coordinated post-discharge care. From a clinical perspective, the post-discharge care requirement after ICU discharge may function as a simple and readily available marker of long-term vulnerability. This information is usually known at the time of discharge and can therefore be incorporated into routine discharge planning. Patients requiring post-discharge care may benefit from early multidisciplinary follow-up, structured rehabilitation, respiratory monitoring, nutritional support, medication review, and caregiver education. In addition, recognizing care requirement as a prognostic marker may help clinicians communicate more realistically with patients and families about expected recovery trajectories and may support more appropriate allocation of post-ICU resources.

Importantly, the inclusion of discharge-phase variables in the multivariable model should be interpreted within a prognostic rather than causal framework. Post-discharge care requirement and spontaneous breathing status may summarize the cumulative effects of pre-existing comorbidity, acute illness severity, respiratory recovery, and functional reserve. Therefore, these variables should be regarded as clinically useful markers for post-ICU risk stratification rather than causal determinants of long-term mortality.

Prior work has emphasized that evaluation of patients aged 80 years and older should incorporate not only clinical stability but also functional capacity and care needs [5]. In the study by Tabah et al. [22], it was reported that only one-third of individuals aged ≥ 80 survived at one year; however, among survivors, most maintained independence in activities of daily living and reported quality of life comparable to the general population. Likewise, in our study, the lower mortality rates among patients who required less post-discharge care and were discharged home—together with the corresponding findings from the multivariable analysis—suggest that these individuals had better premorbid functional capacity, consistent with prior reports.

Significant differences were observed in the post-discharge respiratory status of very elderly ICU survivors. The presence of spontaneous breathing was markedly higher in the survivor group (Table 5). Similarly, spontaneous breathing should be interpreted as a pragmatic marker of respiratory independence at ICU discharge rather than a comprehensive measure of respiratory function or rehabilitation potential. Furthermore, spontaneous breathing was independently associated with reduced one-year mortality in the multivariable model (Table 2). This finding suggests that mortality may be closely related not only to organ failure but also to the level of respiratory independence. Similarly, the need for oxygen concentrators was significantly higher in the non-survivor group (Table 5), indicating more advanced pulmonary dysfunction in these patients.

Corroborating our findings, previous studies have demonstrated that the need for respiratory support is an important factor associated with long-term prognosis in very elderly ICU patients. In patients aged ≥ 80 who required prolonged mechanical ventilation, survival was significantly reduced, and ventilator dependency was identified as an independent risk factor for long-term mortality [23]. Large cohort studies have also shown that respiratory support requirements significantly increase ICU mortality and reduce post-discharge survival rates in patients aged 80 years and older [3].

In the evaluation of feeding methods, oral nutrition was more frequently observed in the survivor group; however, no statistically significant difference was found between the groups (Table 5). A similar pattern was noted for patients receiving nutrition via nasogastric tube or percutaneous endoscopic gastrostomy (PEG). Previous geriatric and critical care studies have reported that patients requiring feeding tubes often have poorer overall condition, greater care needs, and an increased risk of mortality [24,25].

While oral feeding may indicate better functional capacity, it also requires close monitoring due to the increased risk of aspiration and diminished swallowing reflex in elderly individuals. In fact, a study comparing different nutritional methods found that the incidence of aspiration was significantly higher among very elderly ICU patients receiving oral nutrition compared to those receiving enteral feeding [26]. Therefore, although the higher rate of oral feeding observed in the survivor group may be a favorable clinical indicator, it should be carefully evaluated in terms of nutritional safety.

Nevertheless, because patient selection, discharge planning, and post-ICU care organizations may differ across institutions, the present findings should be confirmed in multicenter cohorts before being generalized to broader elderly ICU populations.

### Study Limitations and Strengths

This study has several limitations. First, its single-center and retrospective design limits the generalizability of the findings. In addition, the relatively limited sample size may have reduced the statistical power of the study and restricted the ability to detect weaker associations between several clinical variables and one-year mortality. The relatively small sample size and number of outcome events may also have increased the risk of model overfitting, particularly in multivariable analyses. Although the number of covariates was restricted based on clinical relevance and model performance was evaluated using discrimination, calibration, multicollinearity diagnostics, and bootstrap internal validation, external validation could not be performed. Accordingly, the multivariable model results should be interpreted as exploratory rather than definitive. Larger prospective multicenter studies are needed to validate the prognostic value of post-discharge care requirement, respiratory independence, and comorbidity burden in very elderly ICU survivors.

Because 243 of 394 patients aged 80 years and older died during the ICU stay, corresponding to an ICU mortality rate of 61.7%, the final analytic cohort represents a selected population of ICU survivors. Therefore, the findings cannot be generalized to all very elderly patients admitted to the ICU but should be interpreted as applicable only to patients who survived to ICU discharge. This survivor selection may have influenced the observed associations between post-discharge dependency indicators and one-year mortality.

Baseline functional independence, pre-ICU place of residence, pre-existing care needs, formal cognitive status, and validated frailty scores were not routinely available in the retrospective records. Therefore, post-discharge care requirement may partly represent a surrogate marker of unmeasured pre-existing frailty, cognitive impairment, or baseline dependency rather than solely reflecting dependency newly developed after the ICU stay. Similarly, functional status was not directly measured using validated instruments such as the Barthel Index, Katz Activities of Daily Living scale, or Lawton Instrumental Activities of Daily Living scale. Accordingly, associations involving post-discharge care requirement, discharge destination, respiratory independence, and nutritional route should be interpreted cautiously as pragmatic markers of vulnerability and dependency, not as direct measures of frailty, functional status, or functional decline.

A further limitation is that therapeutic limitations, treatment escalation decisions, and goals-of-care discussions could not be systematically assessed because such decisions were not consistently documented in the retrospective medical records. In very elderly ICU patients, these decisions may influence treatment intensity, discharge destination, post-discharge care requirement, and survival outcomes. Therefore, unmeasured differences in therapeutic goals may have contributed to residual confounding and should be considered when interpreting the observed associations.

Although tracheostomy was recorded as a treatment-related ICU variable, detailed information regarding the indication for tracheostomy, timing of tracheostomy, decannulation status, and long-term tracheostomy dependence was not consistently available in the retrospective records. Therefore, the observed tracheostomy prevalence should be interpreted descriptively, and its relationship with long-term outcomes could not be evaluated in greater detail.

Acute illness severity was assessed using the APACHE II score. Although more contemporary scoring systems such as APACHE IV or SAPS 3 exist, APACHE II remains the routinely used severity score in our institution. Moreover, commonly used ICU severity scores were originally developed to characterize acute-phase illness severity and short-term outcomes, and in the context of the present study, they were interpreted as indicators of baseline illness severity rather than long-term prognostic tools. In addition, the study population was restricted to individuals aged 80 years and older, which prevented comparisons with younger geriatric subgroups and limited insights into age-related effects across the elderly spectrum.

The follow-up period was limited to one year, which did not allow evaluation of longer-term outcomes. Although mortality status was completely ascertained through hospital records and the national death registry, detailed cause-specific mortality data were not consistently available. As a result, the analysis was limited to all-cause one-year mortality, and cause-specific mortality patterns could not be evaluated.

Another important methodological consideration relates to the selection of variables included in the multivariable model. The variables incorporated into the model were determined based on clinical relevance, prior evidence in the literature, and their availability during the ICU stay or at ICU discharge. Post-discharge variables, including post-discharge care requirement and spontaneous breathing status, were included to evaluate their prognostic value for risk stratification after ICU discharge, not to infer causality. Because these variables may partly reflect the consequences of the acute ICU course and may lie on the causal pathway between baseline illness severity and long-term mortality, potential overadjustment bias cannot be excluded. Therefore, associations involving post-discharge variables should be interpreted cautiously as prognostic markers rather than independent causal determinants of mortality. Similarly, the exploratory post-discharge vulnerability indicators were derived from retrospectively available discharge-phase variables and were not based on a previously validated frailty, functional status, or dependency scale. Therefore, they should be interpreted as pragmatic clinical markers rather than validated prognostic tools, and external validation is required.

One of the key strengths of this study is that it is among the few to comprehensively evaluate one-year post-discharge outcomes in ICU patients aged 80 years and older. It not only assessed mortality rates but also included multidimensional clinical indicators such as respiratory support requirements, feeding methods, care needs, and discharge settings. In this respect, the study provides a holistic perspective on both the acute and post-discharge phases of very elderly ICU survivors. This multidimensional post-discharge assessment may be considered a clinically relevant contribution, as it shifts the focus from acute ICU survival alone to the broader vulnerability profile of very elderly patients after ICU discharge. Although the single-center design limits external validity, it also allowed all patients to be managed under relatively standardized clinical protocols and enabled consistent data extraction from the same hospital information system, thereby reducing inter-center variability and measurement heterogeneity.

Given the retrospective observational design, all findings should be interpreted as associations rather than causal effects.

## 5. Conclusions

The primary endpoint of our study was post-discharge survival status among patients aged 80 years and older who survived ICU discharge. The findings indicate that the post-ICU period plays a critical role in prognosis for this age group, with several clinical and discharge-phase factors associated with one-year mortality. These results suggest that improving outcomes in very elderly ICU survivors requires attention not only to the acute care phase but also to post-discharge follow-up, care dependency, respiratory support, and functional rehabilitation. The Charlson Comorbidity Index may help identify ICU survivors at higher risk for one-year mortality, while post-discharge care requirement, non-home discharge destination, and respiratory independence may provide clinically relevant information for risk stratification and follow-up planning after ICU discharge. Very elderly ICU survivors with high comorbidity burden, post-discharge care requirement, non-home discharge destination, or lack of respiratory independence may benefit from structured post-ICU follow-up, multidisciplinary rehabilitation, respiratory and nutritional monitoring, and caregiver support. The data obtained from this study highlight key areas of vulnerability in very elderly ICU survivors and may support the development of more tailored post-discharge follow-up strategies.

## Figures and Tables

**Table 1:** Comparison of Demographic, Clinical, and Score-Based Characteristics of Patients Between Groups.

Parameters	Group 1 Survivors (n = 63)	Group 2 Non-Survivors (n = 68)	*p*-Value
**Age (years), mean ± SD**	84.30 ± 4.26	85.41 ± 3.85	0.038 ^a^
**Sex**			
Female, n (%)	43 (68.3)	36 (52.9)	0.107 ^b^
Male, n (%)	20 (31.7)	32 (47.1)	
**Primary ICU Admission Diagnoses**			
Multiple trauma, n (%)	16 (25.4)	19 (27.9)	0.896 ^b^
Cerebrovascular Pathology, n (%)	21 (33.3)	22 (32.4)	1.000 ^b^
Malignancy, n (%)	7 (11.1)	6 (8.8)	0.885 ^b^
Pulmonary Pathology, n (%)	9 (14.3)	11 (16.2)	0.954 ^b^
Cardiac Pathology, n (%)	6 (9.5)	5 (7.4)	0.895 ^b^
Chronic Organ Failure, n (%)	3 (4.8)	3 (4.4)	1.000 ^b^
Neuromuscular Disease, n (%)	1 (1.6)	2 (2.9)	1.000 ^b^
Charlson Comorbidity Index, mean ± SD	4.08 ± 2.07	5.69 ± 2.27	**<0.001 ^a^**
NRS 2002 score, mean ± SD	1.87 ± 2.07	1.87 ± 1.95	0.996 ^a^
BMI (kg/m^2^), mean ± SD	27.86 ± 3.80	26.41 ± 4.05	**0.044 ^a^**
APACHE II, mean± SD	18.62 ± 7.31	19.57 ± 5.45	0.210 ^a^
SOFA Score, mean ± SD	5.65 ± 2.30	5.99 ± 1.82	0.164 ^a^

n: Number; *p* < 0.05 = Significance level; ICU: Intensive Care Unit; NRS 2002: Nutritional Risk Screening 2002; BMI: Body Mass Index; APACHE II: Acute Physiology and Chronic Health Evaluation II; SOFA: Sequential Organ Failure Assessment Score. ^a^: Mann–Whitney U test, ^b^: Fisher’s Exact test. *p* values which are significant written bold.

**Table 2:** Multivariable Logistic Regression Analysis of Factors Associated with One-Year Mortality.

Variable	OR	95% CI (Lower–Upper)	*p*-Value
Age (years)	1.089	0.967–1.226	0.162
APACHE II Score	1.004	0.924–1.091	0.930
Charlson Comorbidity Index	1.432	1.107–1.851	**0.006**
Mechanical Ventilation (Yes vs. No)	0.471	0.149–1.492	0.201
Vasopressor Use (Yes vs. No)	1.368	0.245–7.645	0.721
Renal Replacement Therapy (Yes vs. No)	0.696	0.087–5.568	0.732
Post-Discharge Care Requirement (Yes vs. No)	6.35	2.83–14.21	<0.001
Spontaneous breathing (Yes vs. No)	0.32	0.15–0.66	0.002

OR: Odds Ratio; CI: Confidence Interval; *p* < 0.05 was considered statistically significant; APACHE II: Acute Physiology and Chronic Health Evaluation II. Significant *p* value bolded.

**Table 3:** Comparison of Clinical Course and Lengths of Stay Between Groups.

Parameters	Group 1 Survivors (n = 63)	Group 2 Non-Survivors (n = 68)	*p*-Value
ICU Length of Stay (days), mean ± SD	11.22 ± 12.47	17.59 ± 19.85	0.059 ^a^
Hospitalization Duration (days), mean ± SD	17.95 ± 15.05	26.00 ± 25.17	**0.043 ^a^**
Mechanical Ventilation, n (%)	26 (41.3)	24 (35.3)	0.590 ^b^
Tracheostomy Performed, n (%)	8 (12.7)	16 (23.5)	0.120 ^b^
ICU-acquired Infection, n (%)	22 (34.9)	26 (38.2)	0.720 ^b^
Vasopressor Use, n (%)	7 (11.1)	20 (29.4)	**0.016 ^b^**
Renal Replacement Therapy, n (%)	2 (3.2)	3 (4.4)	1.000 ^b^
Septic Shock, n (%)	15 (23.8)	22 (32.3)	0.333 ^b^
ICU Readmission, n (%)	14 (22.2)	13 (19.1)	0.673 ^b^

n: Number; *p* < 0.05 = Significance level; ICU: Intensive Care Unit; ^a^: Mann–Whitney U test, ^b^: Fisher’s Exact test. Significant *p* values bolded the other ones corrected.

**Table 4:** Comparison of Patients’ Discharge Characteristics Between Groups.

Parameters	Group 1 Survivors (n = 63)	Group 2 Non-Survivors (n = 68)	*p*-Value
Discharge Destination			
Home Discharge, n (%)	56 (88.9)	47 (69.1)	**0.013 ^b^**
Palliative Care Unit, n (%)	7 (11.1)	18 (26.5)	**0.035 ^b^**
Nursing Home, n (%)	0 (0.0)	3 (4.4)	0.240 ^b^
Post-Discharge Care Requirement			
Care Required, n (%)	11 (17.5)	39 (57.4)	**<0.001 ^b^**
No Care Required, n (%)	52 (82.5)	29 (42.6)	

n: Number; *p* < 0.05 = Significance level; ^b^: Fisher’s Exact test. Post-discharge care requirement refers to documented need for professional or institutional care after ICU discharge. Significant *p* values bolded.

**Table 5:** Comparison of Patients’ Post-Discharge Clinical Characteristics Between Groups.

Parameters	Group 1 Survivors (n = 63)	Group 2 Non-Survivors (n = 68)	*p*-Value
**Respiratory Characteristics**			
Spontaneous Breathing, n (%)	44 (69.8)	29 (42.7)	**0.003 ^b^**
Need for Oxygen Concentrator, n (%)	11 (17.5)	23 (33.8)	**0.046 ^b^**
Home Mechanical Ventilation Dependent, n (%)	8 (12.7)	16 (23.5)	0.120 ^b^
**Feeding Method**			
Oral, n (%)	39 (61.9)	33 (48.5)	0.160 ^b^
Nasogastric, n (%)	6 (9.5)	13 (19.1)	0.141 ^b^
PEG, n (%)	18 (28.6)	22 (32.4)	0.706 ^b^

n: Number; *p* < 0.05 = Significance level; PEG: Percutaneous Endoscopic Gastrostomy; ^b^: Fisher’s Exact test. Spontaneous breathing refers to absence of invasive mechanical ventilation, tracheostomy-dependent ventilation, or home mechanical ventilation support at ICU discharge. Significant *p* values bolded.

**Table 6:** Exploratory Post-Discharge Vulnerability Analysis.

Parameters	Group 1 Survivors (n = 63)	Group 2 Non-Survivors (n = 68)	OR	95% CI	*p*-Value
**Post-discharge care requirement**					
Care required, n (%)	11 (17.5)	39 (57.4)	6.35	2.83–14.21	<0.001
No care required, n (%)	52 (82.5)	29 (42.6)	Reference	—	—
**Discharge destination**					
Non-home discharge, n (%)	7 (11.1)	21 (30.9)	3.57	1.40–9.14	0.010
Home discharge, n (%)	56 (88.9)	47 (69.1)	Reference	—	—

n: Number; OR: Odds Ratio; CI: Confidence Interval. Non-home discharge was defined as discharge to palliative care or nursing home. *p*-values were calculated using Fisher’s exact test. Odds ratios are unadjusted.

## Data Availability

All relevant data are included within the article. Additional information is available from the corresponding author upon reasonable request.
